# Impact of Imaging-Guided Localization on Performance of Tailored Axillary Surgery in Patients with Clinically Node-Positive Breast Cancer: Prospective Cohort Study Within TAXIS (OPBC-03, SAKK 23/16, IBCSG 57-18, ABCSG-53, GBG 101)

**DOI:** 10.1245/s10434-023-14404-4

**Published:** 2023-10-30

**Authors:** Walter P. Weber, Martin Heidinger, Stefanie Hayoz, Zoltan Matrai, Christoph Tausch, Guido Henke, Daniel R. Zwahlen, Günther Gruber, Frank Zimmermann, Giacomo Montagna, Mariacarla Andreozzi, Maite Goldschmidt, Alexandra Schulz, Andreas Mueller, Markus Ackerknecht, Ekaterini Christina Tampaki, Vesna Bjelic-Radisic, Christian Kurzeder, Ákos Sávolt, Viktor Smanykó, Daniela Hagen, Dieter J. Müller, Michael Gnant, Sibylle Loibl, Florian Fitzal, Pagona Markellou, Inga Bekes, Daniel Egle, Jörg Heil, Michael Knauer

**Affiliations:** 1 Breast Center, University Hospital Basel, Basel, Switzerland; 2 University of Basel, Basel, Switzerland; 3 SAKK Competence Center, Bern, Switzerland; 4 Department of Oncoplastic Breast Surgery, Hamad Medical Corporation, Doha, Qatar; 5 Breast Center Zurich, Zurich, Switzerland; 6 Department of Radiation Oncology, St. Gallen Cantonal Hospital, St. Gallen, Switzerland; 7 Breast Center, St. Gallen Cantonal Hospital, St. Gallen, Switzerland; 8 Department of Radiation Oncology, Cantonal Hospital Winterthur, Winterthur, Switzerland; 9 Institute of Radiotherapy, Klinik Hirslanden, Zurich, Switzerland; 10 Clinic of Radiation Oncology, University Hospital Basel, Basel, Switzerland; 11 Breast Service, Department of Surgery, Memorial Sloan Kettering Cancer Center, New York, NY USA; 12 Department of Clinical Research, University Hospital Basel, Basel, Switzerland; 13 Breast Center, Cantonal Hospital Winterthur, Winterthur, Switzerland; 14 Department of Biomedicine, University Hospital Basel, Basel, Switzerland; 15 Department of Plastic, Reconstructive Surgery and Burn Unit, KAT Athens Hospital and Trauma Center, Athens, Greece; 16 Breast Unit, Helios University Clinic, University Witten/Herdecke, Witten, Germany; 17 National Institute of Oncology, Budapest, Hungary; 18 National Tumor Biology Laboratory, National Institute of Oncology, Budapest, Hungary; 19 Bethesda Spital AG, Basel, Switzerland; 20 Comprehensive Cancer Center, Medical University of Vienna, Vienna, Austria; 21 ABCSG, Austrian Breast and Colorectal Cancer Study Group, Vienna, Austria; 22 German Breast Group, GBG Forschungs GmbH, Neu-Isenburg, Germany; 23 Atomos Klinik Waehring, Vienna, Austria; 24 Breast Cancer Center Tirol, Department of Gynecology, Medical University Innsbruck, Innsbruck, Austria; 25 Breast Center Heidelberg, Heidelberg, Germany; 26 Tumor and Breast Center Eastern Switzerland, St. Gallen, Switzerland

**Keywords:** Breast cancer, Breast surgery, Axillary dissection, Sentinel lymph node procedure, Axillary staging

## Abstract

**Background:**

Tailored axillary surgery (TAS) is a novel surgical concept for clinical node-positive breast cancer. It consists of the removal of the sentinel lymph nodes (LNs), as well as palpably suspicious nodes. The TAS technique can be utilized in both the upfront and neoadjuvant chemotherapy (NACT) setting. This study assessed whether/how imaging-guided localization (IGL) influenced TAS.

**Patients and Methods:**

This was a prospective observational cohort study preplanned in the randomized phase-III OPBC-03/TAXIS trial. IGL was performed at the surgeon’s discretion for targeted removal of LNs during TAS. Immediate back-up axillary lymph node dissection (ALND) followed TAS according to TAXIS randomization.

**Results:**

Five-hundred patients were included from 44 breast centers in six countries, 151 (30.2%) of whom underwent NACT. IGL was performed in 84.4% of all patients, with significant variation by country (77.6–100%, *p* < 0.001). No difference in the median number of removed (5 vs. 4, *p* = 0.3) and positive (2 vs. 2, *p* = 0.6) LNs by use of IGL was noted. The number of LNs removed during TAS with IGL remained stable over time (*p* = 0.8), but decreased significantly without IGL, from six (IQR 4–6) in 2019 to four (IQR 3–4) in 2022 (*p* = 0.015). An ALND was performed in 249 patients, removing another 12 (IQR 9–17) LNs, in which a median number of 1 (IQR 0–4) was positive. There was no significant difference in residual nodal disease after TAS with or without IGL (68.0% vs. 57.6%, *p* = 0.2).

**Conclusions:**

IGL did not significantly change either the performance of TAS or the volume of residual nodal tumor burden.

*Trial registration*: ClinicalTrials.gov Identifier: NCT03513614.

**Supplementary Information:**

The online version contains supplementary material available at 10.1245/s10434-023-14404-4.

Axillary surgery has changed remarkably over the last decades. In the 1990s, the sentinel lymph node (SLN) procedure replaced standard axillary lymph node dissection (ALND) as the staging procedure in clinically node-negative patients.^1–3^ Subsequently, the omission of ALND was shown to be oncologically safe in most patients with clinically negative nodes and up to two positive SLNs, which raised interest in the role of modern adjuvant radiotherapy.^4–6^ Surgical de-escalation in the axilla was further extended to involve initially clinically node-positive patients with nodal pathologic complete response (pCR) after neoadjuvant chemotherapy (NACT).^7–11^ In this setting, marking the sampled node with a clip and documentation of its removal was shown to reduce the false-negative rate (FNR) when performing the SLN procedure to determine nodal pCR.^10–16^ Imaging-guided localization (IGL) of the clipped node was introduced to increase the likelihood of clip removal.^17^ The lowest FNR was achieved when IGL was added to the SLN biopsy, a procedure called targeted axillary dissection (TAD).^10,11^ An ALND remains standard in those patients with residual nodal disease after NACT, and in those with palpable axillary disease in the upfront surgical setting.^18,19^

In the ongoing, international, multicenter phase III OPBC-03/TAXIS trial (SAKK 23/16 / IBCSG 57-18 / ABCSG-53 / GBG 101; ClinicalTrials.gov identifier: NCT03513614), patients with clinically node-positive breast cancer undergo tailored axillary surgery (TAS) before being randomized to receive ALND or axillary radiotherapy (ART) in the setting of extended regional nodal irradiation.^20^ While TAS and TAD were both developed to replace ALND, TAS is a therapeutic procedure designed to selectively remove positive nodes, while TAD is a staging procedure designed to selectively remove marked nodes with biopsy-confirmed metastasis before NACT to determine residual nodal disease after NACT.^10,20–23^ This prospective cohort study was pre-planned after 500 randomized patients to investigate the impact of IGL on the performance of TAS in patients with clinically node-positive breast cancer.

## Patients and Methods

This prospective study was preplanned within the randomized controlled international phase-III TAXIS trial (OPBC-03/SAKK 23/16/IBCSG 57-18/ABCSG-53/GBG 101; ClinicalTrials.gov Identifier: NCT03513614). Patients ≥ 18 years of age with histologically or cytologically proven node-positive breast cancer and adequate condition for breast cancer surgery were included. Node-positive disease was detected by palpation or imaging at the time of initial diagnosis. Clip placement in the biopsy-proven positive lymph node was required. Patients were included in both the upfront surgery setting and after NACT in case of residual nodal disease, which was confirmed by either intraoperative frozen section analysis or final histopathology. Patients with stage IV, cN3c or cN2b breast cancer, contralateral or other tumor malignancy within 3 years, prior axillary surgery (except SLN procedure), or prior axillary radiotherapy were considered ineligible. Furthermore, patients with nodal pCR, absence of clip in the specimen radiography, palpable disease left behind in the axilla after TAS, or no SLN identified in the axilla were excluded from randomization. This study was pre-planned after 500 patients were randomized (one-third of the total sample size) in the TAXIS trial. It was planned to gain relevant insight into the impact of IGL on performance of the pragmatic concept of TAS. Patients were treated from August 2018 to June 2022. Data extraction was performed after data cleaning on 30 September 2022.

This study was approved by the local ethics committees and performed in accordance with the requirements of the national regulatory authorities. Written informed consent was obtained from all patients. The study is reported according to the STROBE guidelines (Supplementary Table S1).^24^

### Imaging-Guided Localization (IGL)

Before surgery, the most suspicious axillary node on imaging or palpation was biopsied via core needle biopsy or aspirated with a fine needle according to study-site specific standards and clipped. The clipping was performed at diagnosis or after receiving the pathology report confirming nodal disease. Images of the biopsy and clip placement were documented, e.g., by ultrasound (US). The IGL of the clipped and sampled node was optional, and performance was at the discretion of the surgeon. IGL of radiologically suspicious nodes, in addition to the clipped node, was neither mandatory nor prohibited. Any imaging technology was allowed for IGL, including US, computed tomography (CT), and magnetic resonance imaging (MRI). Any method was allowed for localization of the clipped node, such as the use of wire, iodine-125 or magnetic seeds, radioguided occult lesion localization (ROLL), or a tattoo. Specimen radiography was performed on all removed lymph nodes as measure of surgical quality assurance. If the clip was not documented in the specimen radiography, the patient was not randomized in the TAXIS trial and accordingly, was not included in this study.

### Treatment

The TAS procedure was defined by palpation-guided selective removal of clipped and biopsy proven nodes, SLNs, and removal of all palpably suspicious findings. The sequence of the individual steps, however, was left to the discretion of the surgeon. TAS was designed to turn a clinically node-positive axilla into a clinically negative axilla by removing all palpably obvious disease. TAS could not reflect a procedure as standardized as ALND, because it was designed to de-escalate axillary surgery in a personalized fashion. Therefore, we expected that the pragmatic concept of TAS would result in some degree of intersurgeon variability. During the SLN procedure that was performed according to local standard practice, all nodes that were either blue (blue dye), hot (technetium Tc 99m), fluorescent (indocyanine green), or magnetic (superparamagnetic iron oxide particles) were removed. Dual-tracer mapping was recommended. Patients in the control group of the TAXIS trial underwent TAS followed by back-up ALND after intraoperative randomization in the upfront surgery setting or in case of residual nodal disease when confirmed by intraoperative frozen section analysis. In case of late confirmation of residual disease on final histopathology, randomization was delayed accordingly for a few days. ALND was performed at the discretion of the treating surgeon according to the pragmatic TAXIS trial design and typically cleared levels I and II. A full level III dissection above and medial to the pectoralis minor muscle was only recommended when there was gross nodal disease detected by palpation or imaging. In the experimental arm, patients received TAS followed by axillary radiotherapy (ART). All patients underwent adjuvant whole-breast irradiation after breast-conserving surgery and chest-wall irradiation after mastectomy. While patients in the ALND group received regional nodal irradiation excluding the dissected axilla as a target volume, patients in the ART group received regional nodal irradiation including the axilla.

### Pathologic and Radiologic Evaluation

Pathologic evaluation was not centralized and performed according to the lymph node processing protocol at the local pathology department, thereby adhering to the principles of pragmatism.^25,26^ Nodal pathologic complete response was defined as absence of any nodal disease after NACT, including isolated tumor cells, which, however, were classified as ypN0(i+) according to TNM staging.^27^ Systemic radiologic staging was performed within two months before registration. Repeat staging after NACT was optional. Residual suspicious lymph nodes detected by imaging performed for radiotherapy treatment planning or restaging before the end of adjuvant treatment neither demanded nor prohibited take back surgery for completion ALND or selective removal of these nodes or an additional radiotherapy boost.

### Endpoints

The primary endpoint was the rate of IGL, defined as attempt to selectively localize the clipped node under imaging guidance. Secondary endpoints assessed IGL use by country, year, palpable versus non-palpable disease, and type of clip. Further endpoints investigated the impact of IGL during TAS on number of removed lymph nodes and positive lymph nodes, as well as size of removed lymph node metastasis and rate of residual nodal disease removed during immediate back-up ALND that followed TAS according to the 1:1 randomization in the main TAXIS trial.

### Statistical Analyses

Continuous endpoints were summarized using median and interquartile range (IQR). To assess the influence of covariables, linear regression models were applied that adjusted for potential confounders including upfront surgery versus NACT setting, palpable versus nonpalpable disease, tumor receptor subtype, grade, age, year, and country. Categorical endpoints were summarized using frequency counts and percentages and compared between subgroups of interest using Fisher’s exact test. To assess the influence of covariables on binary variables, logistic regression models were applied. Two-tailed tests with a significance level of 0.05 were used. No adjustment was made for multiple testing and all analyses are considered exploratory. Missing values are reported in Tables 1, 2, 3, and 5. All analyses were performed using *R* (version 4.2.1).

**Table 1:** Patient and tumor characteristics

Variable	No. (%)
No. of patients	500
Age, years	
Median (IQR)	57 (48, 69)
Age, years	
≤ 50	175 (35.0%)
> 50	325 (65.0%)
Neoadjuvant chemotherapy	
No	349 (69.8%)
Yes	151 (30.2%)
Type of breast surgery	
Breast conserving	293 (58.6%)
Mastectomy +/– reconstruction	207 (41.4%)
Tumor size at initial diagnosis, mm	
Median (IQR)	28 (20, 40)
Clinical T stage at initial diagnosis	
T0	2 (0.4%)
T1	117 (23.4%)
T2	292 (58.4%)
T3	59 (11.8%)
T4	24 (4.8%)
Tis (DCIS)	1 (0.2%)
Tx	5 (1.0%)
Clinical N stage at initial diagnosis	
N1 by palpation	226 (45.2%)
N1 by imaging	227 (45.4%)
N2	22 (4.4%)
N3	25 (5.0%)
Postoperative N stage	
pN0	0 (0.0%)
pN1mi	0 (0.0%)
pN1	206 (41.2%)
pN2	85 (17.0%)
pN3	43 (8.6%)
ypN0	1 (0.2%)
ypN0(ITC)	4 (0.8%)
ypN1	122 (24.4%)
ypN2	28 (5.6%)
ypN3	9 (1.8%)
Unknown	2 (0.4%)
Histology	
No special type (NST)	389 (77.8%)
Lobular	60 (12.0%)
Other	50 (10.0%)
Unknown	1 (0.2%)
Receptor status at initial diagnosis	
HR+/HER2−	397 (79.4%)
HR+/HER2+	52 (10.4%)
HR−/HER2+	5 (1.0%)
HR−/HER2−	35 (7.0%)
Missing/unknown	11 (2.2%)
LVI	
Yes	275 (55.1%)
No	223 (44.7%)
Missing/unknown	2 (0.4%)
Modified Bloom–Richardson score	
I	32 (6.4%)
II	294 (58.8%)
III	169 (33.8%)
Missing/unknown	5 (1.0%)

*IQR* interquartile range, *DCIS* ductal carcinoma in situ, *ITC* isolated tumor cells, *HR* hormone receptor, *HER2* human epidermal growth factor receptor 2, *LVI* lymphovascular invasion

**Table 2:** Imaging-guided localization

Variable	No. (%)
Imaging-guided localization of the clipped node: attempted	
Yes	422 (84.4%)
No	78 (15.6%)

	*N* = 422
Imaging-guided localization of the clipped node: successful	
Yes	413 (97.9%)
Unsure	5 (1.2%)
No	4 (0.9%)
Reason for failure (*N* = 4)	
Clip not visible	3 (75.0%)
Wire missed target	1 (25.0%)
Type of clip used to mark the positive node (*N* = 500)	
Direct magseed	36 (7.2%)
Direct radioactive seed	3 (0.6%)
Nitinol ring marker (nickel titanium alloy)	106 (21.2%)
Titanium or stainless-steel marker with gel	129 (25.8%)
Titanium or stainless-steel marker without gel	202 (40.4%)
Other	24 (4.8%)

	*N* = 307
Localization performed before surgery	
Imaging modality used to localize the clipped node (before surgery)	
Ultrasound	301 (98.0%)
Computed tomography	3 (1.0%)
Other	3 (1.0%)
Type of localization used (before surgery)	
Magseed	5 (1.6%)
ROLL	91 (29.7%)
Radioactive seed	31 (10.1%)
Tattoo	6 (2.0%)
Wire	162 (52.9%)
Other	12 (3.9%)

	*N* = 115
Localization performed during surgery	
Type of localization used (during surgery)	
Tattoo	2 (1.7%)
Wire	65 (56.5%)
Ultrasound alone	30 (26.1%)
Other	18 (15.7%)

*ROLL* radioguided occult lesion localization

**Table 3:** Patient and tumor characteristics by use of imaging-guide localization

Variable	No IGL^1^, (*N* = 78)	IGL^1^, (*N* = 422)	*p*-Value^2^
Country			< 0.001
Austria	1 (3.4%)	28 (96.6%)	
Germany	0 (0.0%)	31 (100.0%)	
Hungary	2 (2.0%)	97 (98.0%)	
Italy	0 (0.0%)	2 (100.0%)	
Lithuania	0 (0.0%)	4 (100.0%)	
Switzerland	75 (22.4%)	260 (77.6%)	
Year			< 0.001
2018	0 (0.0%)	20 (100.0%)	
2019	29 (20.1%)	115 (79.9%)	
2020	18 (8.1%)	204 (91.9%)	
2021	9 (16.7%)	45 (83.3%)	
2022	22 (36.7%)	38 (63.3%)	
Method of detection			0.024
Adjuvant, nonpalpable	15 (9.2%)	148 (90.8%)	
Adjuvant, palpable	36 (20.9%)	136 (79.1%)	
Neoadjuvant, nonpalpable	14 (17.7%)	65 (82.3%)	
Neoadjuvant, palpable	13 (15.1%)	73 (84.9%)	
Clinical T stage at initial diagnosis			0.10
cT0	1 (50.0%)	1 (50.0%)	
cT1	24 (20.5%)	93 (79.5%)	
cT2	35 (12.0%)	257 (88.0%)	
cT3	12 (20.3%)	47 (79.7%)	
cT4	5 (20.8%)	19 (79.2%)	
cTis (DCIS)	0 (0.0%)	1 (100.0%)	
cTx	1 (20.0%)	4 (80.0%)	
Postoperative N stage			0.4
pN1	28 (13.6%)	178 (86.4%)	
pN2	17 (20.0%)	68 (80.0%)	
pN3	6 (14.0%)	37 (86.0%)	
Unknown	0 (0.0%)	2 (100.0%)	
ypN0	1 (100.0%)	0 (0.0%)	
ypN0(ITC)	0 (0.0%)	4 (100.0%)	
ypN1	21 (17.2%)	101 (82.8%)	
ypN2	5 (17.9%)	23 (82.1%)	
ypN3	0 (0.0%)	9 (100.0%)	
Tumor type			0.5
NST	59 (15.2%)	330 (84.8%)	
Invasive lobular	8 (13.3%)	52 (86.7%)	
Other	11 (22.0%)	39 (78.0%)	
Unknown	0 (0.0%)	1 (100.0%)	
Tumor grade			0.093
G1	2 (6.2%)	30 (93.8%)	
G2	41 (13.9%)	253 (86.1%)	
G3	35 (20.7%)	134 (79.3%)	
Unknown	0 (0.0%)	5 (100.0%)	
Tumor receptor subtype			0.003
HR+/HER2−	57 (14.4%)	340 (85.6%)	
HR+/HER2+	6 (11.5%)	46 (88.5%)	
HR−/HER2+	2 (40.0%)	3 (60.0%)	
HR−/HER2−	13 (37.1%)	22 (62.9%)	
Unknown	0 (0.0%)	11 (100.0%)	

^1^*n* (%), percentages calculated row-wise

^2^Fisher’s exact test

*IGL* imaging-guided localization, *DCIS* ductal carcinoma in situ, *ITC* isolated tumor cells, *HR* hormone receptor, *HER2* human epidermal growth factor receptor 2

## Results

**Fig. 1:**
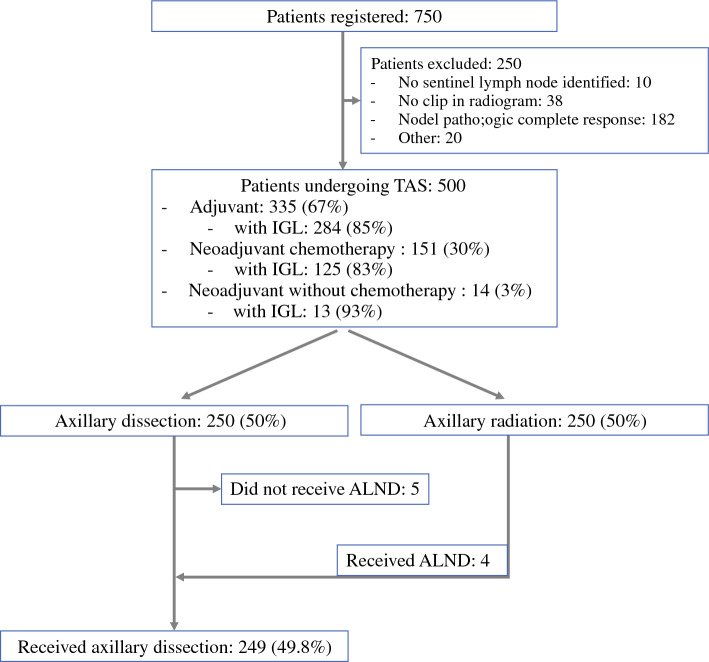
Study flow chart. Reasons for exclusion summarized as “Other”: patient’s refusal of trial treatment, 1; technical problem at site, 1; non-node-positive breast cancer (histologically or cytologically proven both in primary tumor and in lymph node) AJCC/UICC stage II–III, 4; most suspicious axillary lymph node not clipped, 3; baseline quality of life questionnaire not completed, 2; stage IV breast cancer, 1; clinical N3c breast cancer, 1; non-eligible for primary axillary lymph node dissection or sentinel lymph node procedure, 1; palpable disease left behind, 5; contralateral breast cancer within 3 years, 1. *IGL* imaging-guided localization

A total of 500 patients from 44 breast centers were included between August 2018 and June 2022. The median age at diagnosis was 57 years (IQR 48–69 years, Table 1). Of the excluded patients after registration, 72.8% (182/250) had nodal pCR after neoadjuvant treatment, in 4.0% (10/250) no SLN could be identified, in 15.2% (38/250) the clip was not removed during TAS, and 8.0% (20/250) of patients had other exclusion criteria. At the time of initial diagnosis, lymph node metastases were palpable in 51.6% (258/500), and detectable only by imaging in 48.4% (242/500) of patients. Of the 500 randomized patients, 67.0% (335/500) underwent upfront surgery and 30.2% (151/500) had residual nodal disease after NACT, while 2.8% (14/500) received neoadjuvant therapy other than chemotherapy (13 patients received neoadjuvant endocrine therapy; one patient received neoadjuvant double HER2-blockade without chemotherapy). Half of the patients (50%, 250/500) were randomized to undergo ALND—whereas 49.8% (249/500) actually received ALND—and 50% (250/500) to ART (Fig. 1). Of the 165 patients with neoadjuvant treatment, 79.4% (131/165) were randomized intraoperatively after confirmation of residual nodal disease by intraoperative frozen section analysis, whilst 20.6% (34/165) underwent randomization after primary surgery when residual nodal disease was confirmed on final histopathology.

IGL was attempted in 84.4% (422/500) of patients and was considered to have successfully localized the target node in 97.9% (413/422, Table 2). In excluded patients with known IGL status, IGL was attempted in 92.5% (210/227) and successful in 84.3% (177/210, Table S2). The IGL success rate was lowest in excluded patients in which no clip was found (34.4%, 11/32). The most common IGL technique was wire placement (53.8%, 227/422). IGL was used more often for nonpalpable than for palpable disease, with huge variation by country (*p* < 0.001, Table 3). The success of IGL was high with use of US (98%, 295/301). Other imaging modalities used before surgery included CT and MRI in a very limited number of patients (success rate for CT 100%, 3/3; MRI 100%, 3/3). The success of IGL did not depend on type of clip (Table 4). The type of clips used in excluded patients are presented in Table S2. The clip removal rate in all registered patients was 94.9% (712/750). In 74.4% (372/500) of randomized patients, the clipped node corresponded to a SLN. In 128 patients, in which the clipped node was not identified as a SLN, IGL was attempted in 80.5% (103/128), and was considered to have successfully localized the target node in 95.1% (98/103) of these patients.

**Table 4:** Imaging-guided localization by type of clip

	Type of clip					
	Direct magseed	Direct seed	Ring marker	Marker with gel	Marker without gel	*p*-Value^b^
IGL attempted	*N* = 8 *n* (%)	*N* = 2 *n* (%)	*N* = 87 *n* (%)	*N* = 119 *n* (%)	*N* = 188 *n* (%)	
IGL successful^a^	8 (100.0%)	2 (100.0%)	84 (96.6%)	118 (99.2%)	186 (98.9%)	0.4

18 patients had other types of clip

^a^As documented by specimen radiography

^b^Fisher’s exact test, excluding the categories “direct magseed” and “direct seed” due to small sample size

There was no difference in any number of nodes removed by TAS with or without IGL, neither total [5 (IQR 3–8) versus 4 (IQR 3–6), *p* = 0.3], nor positive [2 (IQR 1–4) versus 2 (IQR 1–3), *p* = 0.6], nor negative [2 (IQR 1–4) versus 2 (IQR 0–4), *p* = 0.2, Table 5]. In multivariable linear regression models controlling for confounding variables, the effect of IGL on the number of total as well as positive nodes removed remained nonsignificant (Supplementary Table S3). However, while the number of nodes removed during TAS with IGL remained stable over time [5 (IQR 3–8 in 2019 and 5 (IQR 3–8) in 2022, *p* = 0.8], it decreased significantly without IGL, from 6 (IQR 4–6) in 2019 to 4 (IQR 3–4) in 2022 (*p* = 0.015). The difference in the number of nodes removed by use of IGL was not statistically significant at any time point, neither in 2019 (*p* = 0.6), nor in 2022 (*p* = 0.6). Size of lymph node metastases did not differ by use of IGL (*p* = 0.7).

**Table 5:** Characteristics of tailored axillary surgery by use of IGL

Variable	Upfront surgery *N* = 335		Neoadjuvant chemotherapy* N = 151	
	IGL (*N* = 284)**	No IGL (*N* = 42)**	IGL (*N* = 125)***	No IGL (*N* = 20)***
TAS	Median (IQR)	Median (IQR)	Median (IQR)	Median (IQR)
Total number of nodes removed by TAS	5 (3, 8)	5 (4, 6)	4 (2, 6)	4 (2, 5)
Number of sentinel nodes	3 (2, 4)	2 (1, 4)	3 (2, 4)	2 (1, 3)
Number of palpably suspicious nodes	2 (1, 4)	3 (1, 4)	1 (0, 2)	2 (1, 3)
Number of positive^a^ nodes	2 (1, 4)	2 (1, 3)	1 (1, 2)	2 (1, 3)
Number of negative nodes	2 (1, 4)	2 (0, 4)	2 (1, 4)	1 (0, 3)

	*n*%	*n*%	*n*%	*n*%
Largest sentinel node metastasis				
Isolated tumor cells	0 (0.0%)	0 (0.0%)	6 (4.8%)	0 (0.0%)
Micro	11 (3.9%)	1 (2.4%)	18 (14.4%)	3 (15.0%)
Macro	106 (37.3%)	19 (45.2%)	84 (67.2%)	15 (75.0%)
NA (no positive sentinels)	152 (53.5%)	21 (50.0%)	11 (8.8%)	1 (5.0%)
Unknown	15 (5.3%)	1 (2.4%)	6 (4.8%)	1 (5.0%)

	*n*%	*n*%	*n*%	*n*%
Largest non-sentinel node metastasis				
Isolated tumor cells	1 (0.4%)	0 (0.0%)	1 (0.8%)	0 (0.0%)
Micro	18 (6.3%)	2 (4.8%)	4 (3.2%)	0 (0.0%)
Macro	218 (76.8%)	34 (81.0%)	21 (16.8%)	5 (25.0%)
NA (no positive non-sentinels)	27 (9.5%)	5 (11.9%)	95 (76.0%)	15 (75.0%)
Unknown	20 (7.0%)	1 (2.4%)	4 (3.2%)	0 (0.0%)

ALND	*N* = 142****	*N* = 23	*N* = 61	*N* = 10
	Median (IQR)	Median (IQR)	Median (IQR)	Median (IQR)
Number of additional lymph nodes removed by ALND	13 (9, 18)	12 (10, 18)	12 (9, 15)	12 (8, 14)
Number of additional positive^a,b^ lymph nodes removed by ALND	2 (0, 5)	1 (0, 4)	1 (0, 3)	1 (0, 2)

	*n*%	*n*%	*n*%	*n*%
Number of patients with additional positive^a^ nodes removed by ALND				
No additional positive nodes	42 (30.2%)	9 (39.1%)	24 (39.3%)	5 (50.0%)
One additional positive node	25 (18.0%)	4 (17.4%)	10 (16.4%)	2 (20.0%)
Two additional positive nodes	17 (12.2%)	2 (8.7%)	10 (16.4%)	1 (10.0%)
Three additional positive nodes	8 (5.8%)	2 (8.7%)	3 (4.9%)	0 (0.0%)
Four additional positive nodes	11 (7.9%)	1 (4.3%)	2 (3.3%)	2 (20.0%)
Four additional positive nodes	36 (25.9%)	5 (21.7%)	12 (19.7%)	0 (0.0%)

*IQR* interquartile range, *TAS* tailored axillary surgery, *ALND* axillary lymph node dissection, *NA* not applicable

^a^Nodes with isolated tumor cells are counted as positive

*Fourteen patients had neoadjuvant therapy other than chemotherapy, three of which underwent ALND

**Nine patients for whom IGL was unknown are not shown here, six of which underwent ALND

***Six patients for whom IGL was unknown are not shown here, four of which underwent ALND

****For three patients the number of removed and additional positive lymph nodes was unknown

In the group of patients undergoing ALND after TAS, 4.8% (12/249) presented with bulky lymphadenopathy on clinical examination. Information on additional positive nodes on histopathological examination was available for 246 patients. Immediate back-up ALND following TAS removed another 12 (IQR 9–17) nodes, 1 (IQR 0–4) of which was positive. Greater than or equal to 2 additional positive LNs were found in 47.6% (117/246), and 29.3% (72/246) had ≥ 4 additional positive LNs. The proportion of patients with residual nodal disease in the axilla as assessed by immediate back-up ALND did not differ by use of IGL during TAS (with IGL 68.0%, 136/200; without IGL 57.6%, 19/33; *p* = 0.2). In a multivariable logistic regression model controlling for confounding variables, the effect of IGL on residual nodal disease in patients having undergone ALND remained nonsignificant (Supplementary Table S3).

## Discussion

This is the first prospective study investigating the role of IGL in TAS for clinically node-positive breast cancer in patients undergoing upfront surgery or NACT with residual nodal disease. While IGL was frequently used, it had no impact on the number of positive or negative lymph nodes removed and residual nodal disease after TAS, with two-thirds of patients having at least one additional positive lymph node. Without use of IGL, the number of nodes removed during TAS decreased over the study period, suggesting that surgeons went through a learning curve and sampled fewer nodes at later time points. The study was, however, not designed to assess whether this small difference in number of nodes removed over time was clinically relevant. However, due to the limited impact of IGL on all other performance characteristics of TAS, we conclude that IGL is not a key element of TAS.

TAS consists of palpation-guided selective removal of obvious nodal disease, thereby tailoring the extent of axillary surgery to the extent of axillary disease. It further includes the SLN procedure to reduce the volume of microscopic disease, while IGL of clipped or suspicious nodes is optional.^21^ The underlying hypotheses are that ART can cure microscopic disease left behind in the axilla after TAS in both the neoadjuvant and adjuvant setting, and that the combination of TAS with ART is less harmful for patients compared with standard ALND in the context of extended regional nodal irradiation. Therefore, the new concept of TAS combines several established surgical techniques that are commonly used to stage the axilla in the adjuvant and neoadjuvant setting. While there is no new surgical technique per se involved in TAS, the novel concept is to use a targeted and limited approach to the axilla to treat cancer by selectively removing it, not to rule it out, as is the goal of TAD or the SLN procedure when used in today’s practice.

In the adjuvant setting, the main difference between TAS and the SLN procedure is that TAS allows IGL of nodes, and that the selective removal of palpably suspicious disease is a regular key component. Currently, since palpable disease is the main contraindication to the SLN procedure when used for staging purposes, it is rarely encountered. In the neoadjuvant setting, the main difference between the therapeutic concept of TAS, which serves as final surgical treatment of residual nodal disease, and the diagnostic concept of TAD is that the latter by definition requires IGL and is routinely followed by ALND in case of residual nodal disease.

When planning this study, we were interested to see how the pragmatic concept of TAS would be translated into clinical practice at the participating TAXIS study sites. Therefore, we extensively documented key aspects of TAS including details on preoperative workup (e.g., type of clips and imaging) and the intervention (e.g., number of lymph nodes removed, SLN- and localization techniques). Indeed, significant variation by country was observed. In Switzerland, for example, IGL was omitted in one-quarter of patients, whereas in Austria, Germany, and Hungary, IGL was used in almost all cases (Table 3). International expansion of study sites is currently ongoing. The prespecified evaluation of TAS after randomization of the total TAXIS sample size of 1500 patients will therefore further broaden its global applicability. When use of IGL was attempted, it was successfully performed in 98%. This was not depending on type of clip used (Table 4). However, patients with failed clip removal showed the lowest IGL success rate, suggesting that failed IGL may have increased the risk of missing the targeted node. In our cohort, the clipped node corresponded to an SLN in 74.4% of patients, a finding similar to data published by Kuemmel et al., in which the SLN and targeted lymph node were identical in 64.8%.^13^ Interestingly, IGL was also frequently used for upfront surgery of palpable disease, which reflects the popularity of IGL at some study sites. A prior TAXIS substudy was prespecified after the first 200 patients were randomized and included all details on the 96 patients who were excluded from the TAXIS trial, totaling 296 registered patients.^21^ It showed that the clip removal rate was 94.3% and the false-negative rate of TAS in patients undergoing completion ALND was 1.8%. Since the present substudy focused on use of IGL and its impact on TAS in the first 500 randomized patients, data collection on the 250 excluded patients was limited, and hence, no false-negative rate of TAS could be evaluated. However, since other relevant performance characteristics of TAS remained almost identical, such as the clip removal rate of 94.9%, we have no reason to assume that any of the key findings of the initial substudy have changed after expansion of the patient population.

To the authors’ knowledge, TAXIS is the only RCT addressing the omission of ALND in patients with palpable disease who undergo upfront surgery to date. However, several authors questioned the justification for ALND in this setting by showing low axillary tumor load in many of these patients, and a prospective observational study is currently investigating the selective use of ALND.^28–30^ We expect that further RCTs will soon be developed to investigate the omission of ALND during upfront surgery in light of the results of the RxPONDER and Mindact trials that questioned the routine use of chemotherapy in all patients with luminal node-positive breast cancer.^31,32^

In the neoadjuvant setting, the only RCT besides TAXIS to investigate omission of ALND for residual nodal disease after NACT is the Alliance A011202 trial (NCT01901094). While we eagerly await the primary analysis of this trial, ALND remains standard care, apart from, perhaps, the lowest volume of residual nodal disease, where ART may be considered on an individual basis.^18^ Analyses from the SEER database of patients with residual nodal disease having undergone SLNB and RNI compared with ALND showed conflicting results.^33–35^ Recently, an analysis from the MARI trial, including also patients with residual nodal disease, showed a 3-year axillary recurrence-free interval of 98.2%. Residual disease in the MARI-node independently predicted disease recurrence in multivariable analysis. In this trial, four of five axillary recurrences occurred in patients with residual nodal triple-negative disease and the corresponding 3-year axillary recurrence rate was 3.4%.^36^ We purposefully refrained from performing intrinsic subtype-specific analyses of IGL in TAS for the present substudy due to the limited sample size of non-HR+/HER2− breast cancer (*n* = 103). However, we prespecified the corresponding subgroup analyses in the TAXIS substudy that is planned to assess the performance of TAS after the full TAXIS sample size of 1500 patients is reached. Therefore, the relevance of finding positive nodes in the majority of patients after TAS with 29.3% of patients undergoing ALND having ≥ 4 additional positive nodes, currently remains unclear. Hence, we recommend waiting for oncologic outcome analysis of TAXIS before adopting the much less radical approach of TAS in clinical practice.

### Limitation

This was a prospective observational cohort study embedded in a randomized trial, and IGL was used at the discretion of the surgeon. While IGL was a standard component of TAS in most countries, it was selectively used in Switzerland, where most patients were included. Particularly, the comparison group with no IGL remained rather small (*n* = 78). While we adjusted for multiple confounders including upfront surgery versus NACT setting, palpable versus nonpalpable disease, and tumor receptor subtype in multivariable analyses, residual confounding by unknown factors cannot be excluded.

## Conclusions

TAS targeted positive nodes with or without IGL. IGL proved to be feasible inasmuch as it could be successfully performed in almost all cases, irrespective of type of clip and localization method. However, IGL did not significantly change the performance of TAS, which left ≥ 2 positive nodes behind in 47.6% of patients.

### Supplementary Information

Below is the link to the electronic supplementary material.

Supplementary file1 (DOCX 39 kb)
