# Supplementary material for: Impact of Imaging-Guided Localization on Performance of Tailored Axillary Surgery in Patients with Clinically Node-Positive Breast Cancer: Prospective Cohort Study Within TAXIS (OPBC-03, SAKK 23/16, IBCSG 57-18, ABCSG-53, GBG 101)
Source: Ann Surg Oncol. 2023 Oct 30;31(1):344–55. doi: 10.1245/s10434-023-14404-4 (PMC10695869; doi:10.1245/s10434-023-14404-4)
Supplement: Supplementary file 1 — Supplementary file1 (DOCX 39 kb) [file 10434_2023_14404_MOESM1_ESM.docx]

**Supplementary Material**

**Impact of imaging-guided localization on performance of tailored axillary surgery in patients with clinically node-positive breast cancer: Prospective cohort study within TAXIS (OPBC-03, SAKK 23/16, IBCSG 57-18, ABCSG-53, GBG 101)**

Walter P. Weber, MD^1,2,*,†^; Martin Heidinger, MD^1,2,†^; Stefanie Hayoz, PhD^3^; Zoltan Matrai, MD, PhD^4^; Christoph Tausch, MD^2,5^; Guido Henke, MD^6,7^; Daniel R. Zwahlen, MD^8^; Günther Gruber, MD^9^; Frank Zimmermann, MD^2,10^; Giacomo Montagna, MD, MPH^11^; Mariacarla Andreozzi, PhD^1,2^; Maite Goldschmidt^1,2^; Alexandra Schulz^2,12^; Andreas Mueller, MD^3,13^; Markus Ackerknecht, PhD^2,14^; Ekaterini Christina Tampaki, MD, PhD^15^; Vesna Bjelic-Radisic, MD^16^; Christian Kurzeder, MD^1,2^; Ákos Sávolt MD, PhD^17^; Viktor Smanykó, MD^18^; Daniela Hagen, MD^13^; Dieter J. Müller, MD^19^; Michael Gnant, MD^20,21^, Sibylle Loibl, MD^22^; Florian Fitzal, MD^21,23^; Pagona Markellou, MD^7^; Inga Bekes, MD^7^; Daniel Egle, MD^21,24^; Jörg Heil, MD^25^ and Michael Knauer, MD, PhD^26^

† Contributed equally

^1^ Breast Center, University Hospital Basel, Basel, Switzerland

^2^ University of Basel, Basel, Switzerland

^3^ SAKK Competence Center, Bern, Switzerland

^4^ Hamad Medical Corporation, Dept of Oncoplastic Breast Surgery, Doha, Qatar

^5^ Breast Center Zurich, Zurich, Switzerland

^6^ Department of Radiation Oncology, St. Gallen Cantonal Hospital, St. Gallen, Switzerland

^7^ Breast Center, St. Gallen Cantonal Hospital, St. Gallen, Switzerland

^8^ Department of Radiation Oncology, Cantonal Hospital Winterthur, Winterthur, Switzerland

^9^ Institute of Radiotherapy, Klinik Hirslanden, Zurich, Switzerland

^10^ Clinic of Radiation Oncology, University Hospital Basel, Basel, Switzerland

^11^Breast Service, Department of Surgery, Memorial Sloan Kettering Cancer Center, New York, NY, USA

^12^Department of Clinical Research, University Hospital Basel, Basel, Switzerland

^13^ Breast Center, Cantonal Hospital Winterthur, Winterthur, Switzerland

^14^ Department of Biomedicine, University Hospital Basel, Basel, Switzerland

^15^ Department of Plastic, Reconstructive Surgery and Burn Unit, KAT Athens Hospital and Trauma Center, Athens, Greece

^16^ Breast Unit, Helios University Clinic, University Witten/Herdecke, Germany

^17^ National Institute of Oncology, Budapest, Hungary

^18^ National Tumor Biology Laboratory, National Institute of Oncology, Budapest, Hungary

^19^ Bethesda Spital AG, Basel, Switzerland

^20^ Comprehensive Cancer Center, Medical University of Vienna, Vienna, Austria

^21^ ABCSG, Austrian Breast and Colorectal Cancer Study Group, Vienna, Austria

^22^ German Breast Group, GBG Forschungs GmbH, Neu-Isenburg, Germany

^23^ Atomos Klinik Waehring, Vienna, Austria

^24^ Breast Cancer Center Tirol, Department of Gynecology, Medical University Innsbruck, Innsbruck, Austria

^25^ Breast Center Heidelberg, Heidelberg, Germany

^26^ Tumor and Breast Center Eastern Switzerland, St. Gallen, Switzerland

*** Corresponding Author:** Prof. Walter P. Weber, Breast Center, University Hospital Basel, Spitalstrasse 21, 4031 Basel, Switzerland; e-mail: walter.weber@usb.ch; phone: +41 61 328 61 49.

**Table S1. STROBE Statement**

|  | | Item No | Recommendation | Page No |
| --- | --- | --- | --- | --- |
| **Title and abstract** | | 1 | (*a*) Indicate the study’s design with a commonly used term in the title or the abstract | 1 |
|  |  |  | (*b*) Provide in the abstract an informative and balanced summary of what was done and what was found | 7-8 |
| Introduction | | | | |
| Background/rationale | | 2 | Explain the scientific background and rationale for the investigation being reported | 9 |
| Objectives | | 3 | State specific objectives, including any prespecified hypotheses | 10 |
| Methods | | | | |
| Study design | | 4 | Present key elements of study design early in the paper | 10 |
| Setting | | 5 | Describe the setting, locations, and relevant dates, including periods of recruitment, exposure, follow-up, and data collection | 10 |
| Participants | | 6 | (*a*) Give the eligibility criteria, and the sources and methods of selection of participants. Describe methods of follow-up | 10 |
|  |  |  | (*b*) For matched studies, give matching criteria and number of exposed and unexposed |  |
| Variables | | 7 | Clearly define all outcomes, exposures, predictors, potential confounders, and effect modifiers. Give diagnostic criteria, if applicable | 11-13 |
| Data sources/ measurement | | 8* | For each variable of interest, give sources of data and details of methods of assessment (measurement). Describe comparability of assessment methods if there is more than one group | 10-13 |
| Bias | | 9 | Describe any efforts to address potential sources of bias | 10-13 |
| Study size | | 10 | Explain how the study size was arrived at | 10 |
| Quantitative variables | | 11 | Explain how quantitative variables were handled in the analyses. If applicable, describe which groupings were chosen and why | 10-13 |
| Statistical methods | | 12 | (*a*) Describe all statistical methods, including those used to control for confounding | 13 |
|  |  |  | (*b*) Describe any methods used to examine subgroups and interactions |  |
|  |  |  | (*c*) Explain how missing data were addressed |  |
|  |  |  | (*d*) If applicable, explain how loss to follow-up was addressed |  |
|  |  |  | (*e*) Describe any sensitivity analyses |  |
| Results | | | |  |
| Participants | | 13* | (a) Report numbers of individuals at each stage of study—eg numbers potentially eligible, examined for eligibility, confirmed eligible, included in the study, completing follow-up, and analysed |  |
|  |  |  | (b) Give reasons for non-participation at each stage | 14-15; Figure 1 |
|  |  |  | (c) Consider use of a flow diagram |  |
| Descriptive data | | 14* | (a) Give characteristics of study participants (eg demographic, clinical, social) and information on exposures and potential confounders | 14-15; Tables 1, 2, 3, 5 |
|  |  |  | (b) Indicate number of participants with missing data for each variable of interest |  |
|  |  |  | (c) Summarise follow-up time (eg, average and total amount) |  |
| Outcome data | | 15* | Report numbers of outcome events or summary measures over time | 14-16 |
| Main results | 16 | (*a*) Give unadjusted estimates and, if applicable, confounder-adjusted estimates and their precision (eg, 95% confidence interval). Make clear which confounders were adjusted for and why they were included | | 14-16 |
|  |  | (*b*) Report category boundaries when continuous variables were categorized | |  |
|  |  | (*c*) If relevant, consider translating estimates of relative risk into absolute risk for a meaningful time period | |  |
| Other analyses | 17 | Report other analyses done—eg analyses of subgroups and interactions, and sensitivity analyses | | 14-16 |
| Discussion | | | | |
| Key results | 18 | Summarise key results with reference to study objectives | | 16-19 |
| Limitations | 19 | Discuss limitations of the study, taking into account sources of potential bias or imprecision. Discuss both direction and magnitude of any potential bias | | 19-20 |
| Interpretation | 20 | Give a cautious overall interpretation of results considering objectives, limitations, multiplicity of analyses, results from similar studies, and other relevant evidence | | 20 |
| Generalisability | 21 | Discuss the generalisability (external validity) of the study results | | 16-20 |
| Other information | | | | |
| Funding | 22 | Give the source of funding and the role of the funders for the present study and, if applicable, for the original study on which the present article is based | | 3 |

**Table S2. Characteristics of IGL in excluded patients**

|  | **Total excluded patients (N=227^1^)** | **Excluded patients with nodal pCR (N=168^1^)** | **Excluded patients other than nodal pCR (N=59^1^)** | **p-value^2^** |
| --- | --- | --- | --- | --- |
| IGL attempted | 210 (92.5%) | 155 (92.3%) | 55 (93.2%) | 0.8 |
| IGL successful | 177 (84.3%) | 144 (92.9%) | 33 (60.0%) | <0.001 |
| Type of clip used | N= 227 | N=168 | N=59 | 0.2 |
| Direct Magseed | 8 (3.5%) | 7 (4.2%) | 1 (1.7%) |  |
| Nitinol ring marker (nickel titanium alloy) | 65 (28.6%) | 49 (29.2%) | 16 (27.1%) |  |
| Titanium or stainless steel marker with gel | 63 (27.8%) | 41 (24.4%) | 22 (37.3%) |  |
| Titanium or stainless steel marker without gel | 80 (35.2%) | 62 (36.9%) | 18 (30.5%) |  |
| Other | 11 (4.8%) | 9 (5.4%) | 2 (3.4%) |  |
| Reasons for failed IGL | N=27^3^ | N=6 | N=21 | 0.10 |
| Clip not visible | 21 (77.8%) | 3 (50.0%) | 18 (85.7%) |  |
| Close to vessel | 1 (3.7%) | 1 (16.7%) | 0 (0.0%) |  |
| Other | 5 (18.5%) | 2 (33.3%) | 3 (14.3%) |  |

^1^ Patients with unknown IGL status (n=23) were excluded

^2^ Fisher's exact test

^3^ In 6 patients IGL success was unsure

**Table S3. Linear regression model for the effect of imaging-guided localization on the number of total and positive nodes removed and logistic regression model for the effect on residual nodal disease in the axilla after TAS.**

|  | Unadjusted | | | Adjusted | | |
| --- | --- | --- | --- | --- | --- | --- |
|  | Coefficient | 95% CI | p-value | Coefficient | 95% CI | p-value |
| Total number of lymph nodes removed by TAS | 0.55 | -0.61 - 1.71 | 0.349 | 0.69 | -0.53 - 1.90 | 0.267 |
| Number of positive lymph nodes removed by TAS | 0.30 | -0.51 - 1.11 | 0.468 | 0.44 | -0.40 - 1.28 | 0.306 |
|  | Odds ratio | 95% CI | p-value | Odds ratio | 95% CI | p-value |
| Residual nodal disease after TAS | 0.61 | 0.31 - 1.19 | 0.146 | 0.73 | 0.34 - 1.57 | 0.414 |

Multivariable analyses adjusted for upfront surgery vs neoadjuvant chemotherapy, palpable vs non-palpable disease, tumor receptor subtype, grade, age, year and country. The group without use of imaging-guided localization was defined as the reference group.
